# Morphological and Morphometric Analysis of Superior Articular Facet of Atlas Vertebra

**DOI:** 10.7759/cureus.22906

**Published:** 2022-03-06

**Authors:** Tejaswi H Lokanathan, Ajay Ningaiah, Shanthigrama K Asharani, Yogi A Balakrishnan, Shilpashree Y Dhananjaya

**Affiliations:** 1 Anatomy, Adichunchanagiri Institute of Medical Sciences, Nagamangala, IND; 2 Biochemistry, Adichunchanagiri Institute of Medical Sciences, Nagamangala, IND

**Keywords:** morphology, variations, atlanto-axial joint, superior articular facet, atlas, humans

## Abstract

Introduction: The first cervical vertebra, also referred to as the atlas, forms a vital part of the craniovertebral junction (CVJ). The anatomy of the atlas is essential to understand the basis for CVJ anomalies and their surgical correction. The present study was undertaken to provide accurate morphometry and describe the morphological variations of the superior articular facet (SAF) of the atlas.

Methods: In this observational, cross-sectional study, the length and width of the SAF of 150 atlas were measured using a digital caliper. The variations in the morphology of the SAF in the same bones were also recorded. Z-test was applied to find the statistically significant difference between the measurements of the SAF of the atlas on the right and left sides. p-value ≤ 0.05 was considered statistically significant.

Results: The width of the SAF of the atlas was found to be significantly (p < 0.001) greater on the left side (8.76 mm to 14.84 mm) compared to the right (7.67 mm to 14.83 mm). The mean length of the SAF was 21.1 mm and 21.9 mm on the left and right sides, respectively. Morphologically, four variations of superior articular facets were noted: oval, bilobed, kidney, and dumbbell shaped. The most common variation was an oval-shaped facet with a prevalence of 66.7% on the left side and 57.3% on the right. The least common variation was the bilobed facet, with a prevalence of 4% on the left side and 8.7% on the right.

Conclusion: The width of the SAF of the atlas was statistically significant on the left side. Morphologically, four types of variations were observed in the shape of the SAF. Knowledge of the morphological and morphometric variations of the SAF can be of help during surgical approaches at the CVJ.

## Introduction

Atlas bone, or the first cervical vertebra (C1), forms the vital link between the skull and the vertebral column and includes a transition zone between a mobile cranium and rigid vertebral column [1]. The atlas comprises two lateral masses connected by a short anterior and a longer posterior arch. The lateral masses have a kidney-shaped superior articular facet (SAF) and a round- or oval-shaped inferior articular facet (IAF) [2]. The SAF articulates superiorly with the occipital condyles of the skull, forming the atlantooccipital joint. The inferior articular facet articulates with the axis vertebra [2].

The craniovertebral joint (CVJ) between the superior articular facets of the first cervical vertebra and occipital condyles of the skull is an essential subject of study. The shape and size of the SAF of the atlas are imperative contributing factors in the stability of CVJ [3]. Alterations in the morphology of the SAF can lead to instability at the CVJ leading to restrictions of the movements or hypermotility at the CVJ resulting in vascular and neurological symptoms [4].

The alterations in the morphology of the SAF can be due to several causes, congenital, traumatic, chronic inflammatory disorders, degenerative, or infections. The altered morphology of the atlas can result in instability at the CVJ that warrants surgical correction. The instability at the CVJ is often treated by various surgical fixation techniques [5,6]. These surgical techniques require an accurate understanding of the anatomy of the SAF of the atlas.

Literature review reveals multiple studies reporting the anatomical variations in the morphology and morphometry of atlas [7-9]. In most of these previous studies, the sample size studied is relatively small, which necessitates a need for a detailed morphometric and morphological analysis of the SAF using a larger sample size [7-10]. The present research was undertaken to add to the existing knowledge by providing accurate measurements and describing the variations in the morphology of various dimensions of SAF of the atlas of South Indian origin.

## Materials and methods

This observational, cross-sectional study was conducted on 150 dry, adult, human atlas bones of unspecified sex randomly collected from the Department of Anatomy of a medical college in South India. The sample size was arrived at using the formula n = (Zα)2σ2/L2 (Zα = 1.96 at 95% confidence interval, σ = standard deviation, and L = allowable error of 1%). The study included 150 fully ossified and processed atlas bones. Unossified bones with or without defects were excluded from the study. 

**Figure 1:**
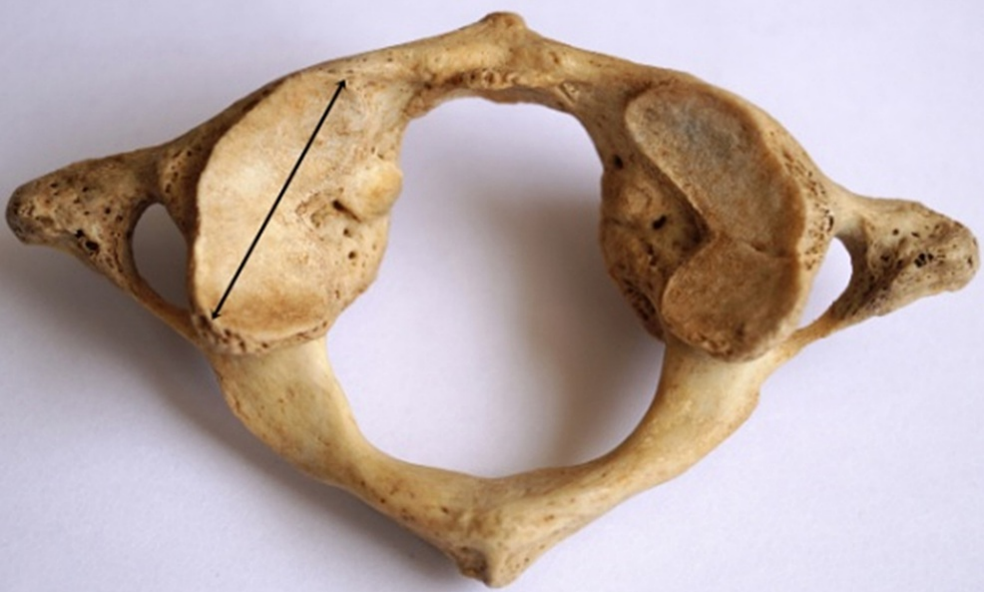
Measurement of the length of SAF Black double-headed arrow indicates the maximum anteroposterior dimension/length of the SAF. SAF: superior articular facet.

**Figure 2:**
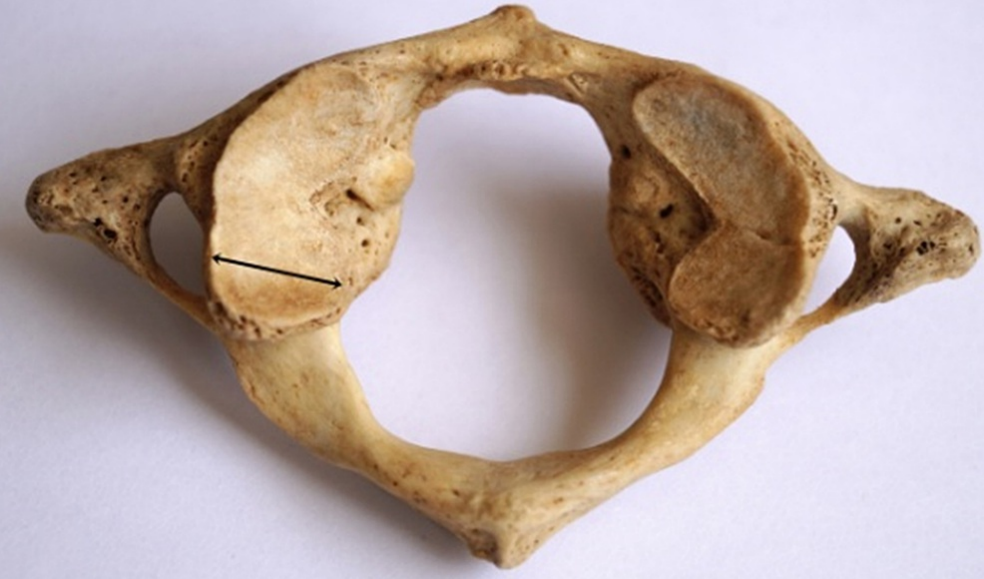
Measurement of the width of SAF Black double-headed arrow indicates the maximum transverse dimension/width of the SAF. SAF: superior articular facet.

The primary author collected the data by measuring the length and width of the SAF of atlas using a digital caliper and observing the SAF's shape. Each measurement was done thrice, and the average was considered the final reading. The length was measured as the maximum anteroposterior diameter of the SAF (Figure 1), and the width was measured as the maximum transverse diameter of the SAF (Figure 2). All measurements done were following reference points described by Sengul and Kadioglu [10]. 

The results were expressed as mean, range, and standard deviation for measurements of SAF of the atlas. Z-test was applied to find the statistically significant difference between the measurements of the SAF of the atlas on the right and left sides. p-value ≤ 0.05 was considered statistically significant. The shape of the SAF was grouped into four types based on observations by Lalit et al. [11]. The SAF with no constrictions was considered oval shaped; SAF with constriction on one side of the facet was considered kidney shaped; SAF with constrictions on both sides of the facet was considered dumbbell shaped; and SAF with complete separation of facets into two distinct segments was deemed to be bilobed. 

## Results

The range, mean, and standard deviation of length of the SAF of the atlas are mentioned in Table 1. The mean length of the SAF was 21.99 mm and 22.09 mm on the right and left sides, respectively. The difference in the length of the SAF on the right and left sides was not statistically significant.

**Table 1:** The range, mean, and standard deviation of length of the SAF of atlas SAF: superior articular facet.

Number of bones	Side	Range (mm)	Minimum (mm)	Maximum (mm)	Mean (mm)	Standard deviation	p-value
150	Right	10.85	17.84	28.69	21.9954	2.07525	0.653
	Left	9.87	17.90	27.77	22.0991	1.91140	

Table 2 shows the range, mean, and standard deviation of the width of the SAF of the atlas. The mean width of the SAF was 10.78 mm on the right side and 11.23 mm on the left. The difference in the width of SAF on the two sides was statistically significant (p = 0.006) (Table 2).

**Table 2:** The range, mean, and standard deviation of width of the SAF of atlas SAF: superior articular facet.

Number of bones	Side	Range (mm)	Minimum (mm)	Maximum (mm)	Mean (mm)	Standard deviation	p-value
150	Right	7.16	7.67	14.83	10.7897	1.52332	0.006
	Left	6.06	8.76	14.84	11.2380	1.29769	

**Figure 3:**
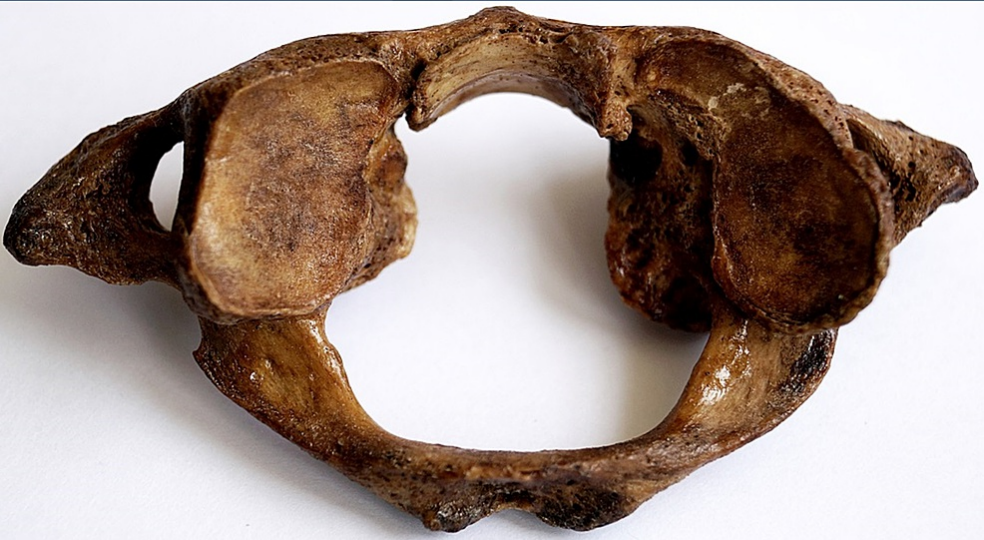
Oval-shaped SAF on the left side SAF: superior articular facet.

**Figure 4:**
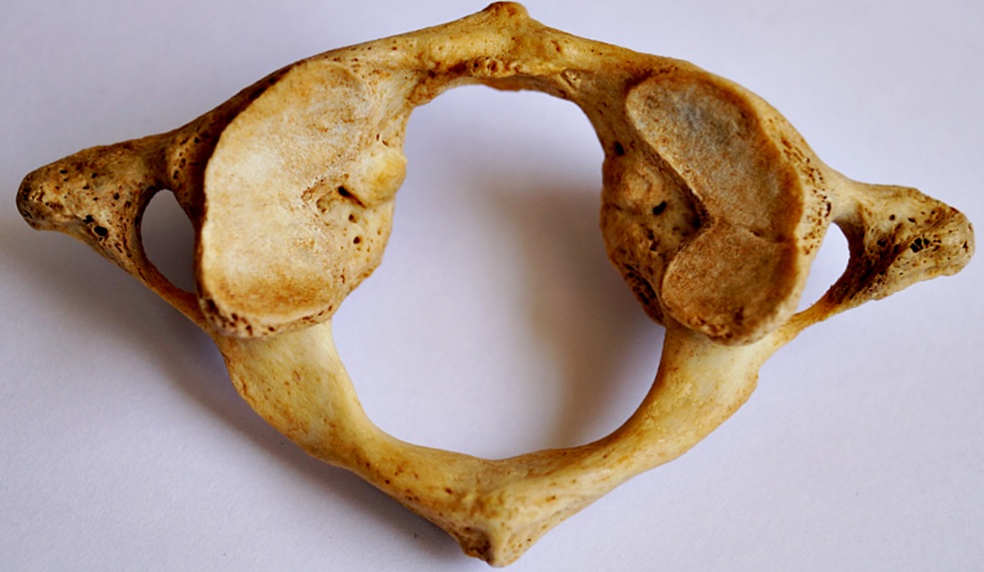
Bilateral kidney-shaped SAF SAF: superior articular facet.

**Figure 5:**
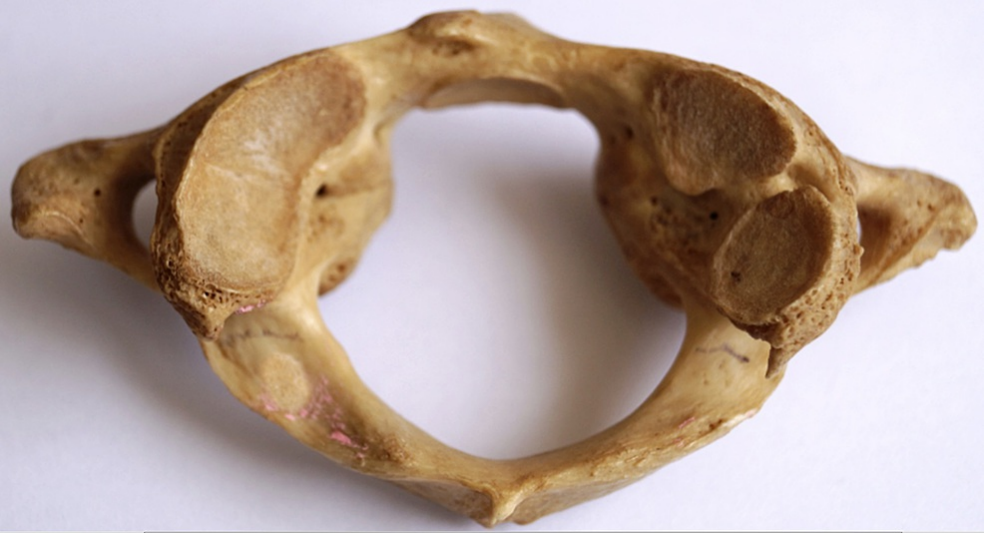
Kidney-shaped SAF on the left side and bilobed SAF on the right side SAF: superior articular facet.

**Figure 6:**
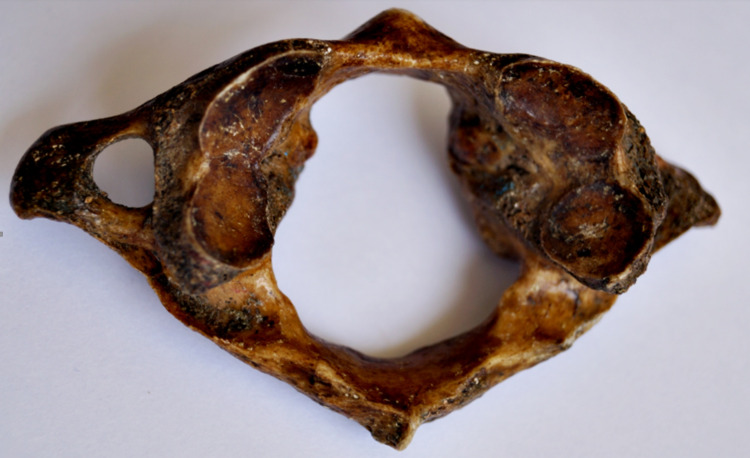
Dumbbell-shaped SAF on the left side and bilobed SAF on right side SAF: superior articular facet.

The shape of the superior articular facet varied considerably (Table 3). Four types of SAF were identified: oval (Figure 3), kidney shaped (Figure 4), bilobed (Figure 5), and dumbbell shaped (Figure 6). The most common type was oval-shaped SAF (57.3% right and 66.7% left). The least common type was the bilobed superior articular facet, with a prevalence of 8.7% on the right side and 4% on the left side. Inferior articular facets were circular and showed minimal variation.

**Table 3:** Prevalence of different shapes of SAF SAF: superior articular facet.

Number of bones	Side	Oval	Kidney	Bilobed	Dumbbell
150	Right	86 (57.3%)	29 (19.3%)	13 (8.7%)	22 (14.7%)
	Left	100 (66.7%)	25 (16.7%)	6 (4%)	19 (12.7%)

## Discussion

Morphology and morphometry of the SAF of the atlas are essential to understand the anatomical basis for the CVJ abnormalities as well as its surgical fixation. While morphometry of the SAF is crucial in transpedicular screw, in our present study, we measured the dimensions of the SAF. We observed the morphological types and variations in the shape of the SAF of the dry atlas bone of possibly the South Indian population.

We found a statistically significant difference in the width of the SAF between the right and left sides. However, the length of the SAF did not differ significantly on the right and left sides. We also found four distinct types of SAF: oval, kidney, bilobed, and dumbbell. Table 4 shows that the length and width of SAF of the atlas in the Indian population are slightly higher than the measurements in Turkish and German people but marginally lower than that in the US population [10,12-16].

**Table 4:** Comparison of length and width of SAF of the atlas with other studies SAF: superior articular facet, NA: not available.

Studies	No. of atlas studied	Place of study	Mean length of the superior articular facet (mm)		Mean width of the superior articular facet (mm)	
			Right	Left	Right	Left
Sengul and Kadioglu [10]	40	Turkey	19.9	18.6	9.6	9.8
Gosavi and Vatsalaswamy [12]	100	India	21.24	21.02	10.36	10.47
Gupta et al. [13]	35	India	21.5	21.8	11.8	11.5
Konig et al. [14]	30	Germany	19.1	18.7	11.6	11.2
Kaur et al. [15]	50	India	21.52	21.51	11.21	11.32
Rocha et al. [16]	20	USA	23.9	23.6	NA	NA
Present study	150	India	21.9954	22.0991	10.7897	11.2380

Table 5 shows the comparison of the shape of SAF of the atlas with other published studies. Various authors have divided the SAF into oval-shaped, kidney-shaped, dumbbell-shaped or figure of 8 shaped, bilobed, irregular, and leaf-like facets. In the present study, the most common shape of SAF was oval (62%), followed by kidney-shaped facets (18%). The present study's findings align with other authors except for Lalit et al., Motagi and Ranganath, and Gupta et al. [4,11,13]. The three studies mentioned above describe the dumbbell shape or figure of 8 shapes as the most typical type of SAF. This difference could be due to the number of specimens studied and environmental factors [11]. The difference in the shape of SAF can be due to the functional modification because of the erect posture of human beings [4]. As we can appreciate from Table 4, race of the population has a trivial effect on the shape of SAF.

**Table 5:** Comparison of shape of SAF of the atlas with other studies SAF: superior articular facet.

Studies	No. of atlas studies	Place of study	Shape of the -superior articular facet					
			Oval	Kidney	Dumbbell	Bilobed	Irregular	Leaf like
Sengul and Kadioglu [10]	40	Turkey	72%	28%	0	0	0	0
Gosavi and Vatsalaswamy [12]	100	India	74%	26%	0	0	0	0
Gupta et al. [13]	35	India	42.8%	7.1%	31.4%	0	0	18.7%
Lalit et al. [11]	30	India	23.3%	20%	56.7%	0	0	0
Motagi and Ranganath [4]	50	India	33%	10%	18%	10%	39%	0
Cacciola et al. [17]	20	India	76%	24%	0	0	0	0
Present study	150	India	62%	18%	13.7%	6.35%	0	0

The stability of the atlantooccipital joint depends on the configuration of the occipital condyles with SAF. The orientation of SAF of the atlas is horizontal during development and becomes concave by six years of development. With advancing age, morphology and morphometry of SAF may vary, causing asymptomatic or symptomatic clinical conditions [4]. The appearance of dumbbell-shaped or a bilobed SAF indicates the tendency of the SAF to split into two separate facets, and it can cause restriction in the movement of the atlantooccipital joint [17].

One of the limitations of our study was that we did not measure the parameters of the SAF of atlas using CT scans. We wish to further our research by measuring the dimensions of the atlas in living and correlating the same with the present study's findings. Furthermore, the results obtained are specific to South Indian origin, and hence generalizing the study results requires further research in different ethnicity and geographical area.

## Conclusions

An increase in the number and innovations in the surgical approach to the CVJ calls for a detailed morphometric analysis of the atlas vertebra. This observational study revealed that the width of SAF of the atlas is significantly higher on the left side than on the right side. The study also showed four morphological types of SAF, and the oval-shaped SAF was the most typical type. The strength of the present study is the number of samples studied compared to the previous researchers. The present study's findings may be helpful for anatomists for academic purposes and the clinicians who perform surgical procedures at the CVJ to anticipate and minimize complications. 
